# Inverse focusing inside turbid media by creating an opposite virtual objective

**DOI:** 10.1038/srep29452

**Published:** 2016-07-11

**Authors:** Yeh-Wei Yu, Szu-Yu Chen, Che-Chu Lin, Ching-Cherng Sun

**Affiliations:** 1 Department of Optics and Photonics, National Central University, Chung-Li, Taoyuan City, 32001 Taiwan; 2 Optical Science Center, National Central University, Chung-Li, Taoyuan City, 32001 Taiwan

## Abstract

Limited by the penetration depth, imaging of thick bio-tissues can be achieved only by epi-detection geometry. Applications based on forward-emitted signals or bidirectional illumination are restricted by lack of an opposite objective. A method for creating an opposite virtual objective inside thick media through phase conjugation was first proposed. Under forward illumination, the backward scattering light from the media was collected to generate a phase conjugate wave, which was sent back to the media and formed an inverse focusing light. Samples combined with a diffuser or a mouse skin were used as specimens. Inverse focusing was successfully demonstrated by applying holography-based optical phase conjugation with a BaTiO_3_. This result indicates the capability to create an opposite virtual objective inside live tissues. The proposed method is compatible with current coherent imaging and super-resolution imaging technologies. It creates a possible way for forward-emitted signals collection and bidirectional illumination in thick specimens.

Optical microscopy is applicable to a variety of biomedical applications because of its high spatial resolution, low photodamage, and high acquisition rate. The invention of virtual biopsy techniques, such as confocal^1^ and multiphoton microscopy^2^, have further progressed thick tissue and *in vivo* investigation by increasing the axial resolution. For intact or live optically thick tissues, epi-detection is a commonly used geometry. An objective is used for both illumination and detection. However, without an opposite objective, signals that can be epi-detected are limited to directly backward-emitted signals such as fluorescence and backward-scattered signals. The relative intensity of backward- and forward-emitted fluorescence depends on the fluorophore particle size^3^,^4^. Large particles have stronger backward fluorescence, whereas small particles prefer forward fluorescence. Harmonic generation signals that prefer forward emission^5^,^6^ can be detected only through the backward scattering of surrounding tissues. The detection efficiency of backward harmonic generation signals is much lower than that of forward signals, limiting the imaging depth^7^,^8^,^9^,^10^. Affected by the scattering process, the information carried by backward and forward harmonic generation signals has been proven to be different^11^,^12^,^13^,^14^,^15^,^16^. To increase the collection efficiency or to obtain the information carried by forward harmonic generation signals in thick tissues, backward illumination is required. However, the epi-detection geometry rules out applications that require bidirectional illumination or detection. For instance, 4-pi microscopy involves applying bidirectional illumination to create an axial interference fringe to improve the axial resolution^17^. All 4-pi-related techniques such as 4-pi-stimulated emission depletion (STED) microscopy^18^,^19^,^20^, 4-pi confocal microscopy^17^, and 4-pi trapping^21^,^22^,^23^ require two opposite objectives at each side of the observed tissues. Therefore, their applications in optically thick tissues have never been proposed without backward illumination generated inside the tissue.

In this paper, we propose a novel concept of generating an opposite virtual objective (OV-Obj) (Fig. 1). The OV-Obj serves as an objective at the other side of the observed tissue that generates the desired backward illumination inside the tissue. The concept is stated as follows. A physical objective (P-Obj) is used to forward focus a laser beam in the tissue and to collect the backward scattering light from the tissue. The backward scattering light is sent to the holography-based optical phase-conjugate mirror (HOPCM) to generate a phase conjugate wave. When the phase conjugate wave illuminates the tissue, the tissue is activated to form an inverse focusing light. This process is regarded as an OV-Obj. Under this configuration, the forward and inverse focusing beams not only focus at the same point but also inherently possess the same phase at the focal plane^24^. This autopositioning and auto-phase-matching can benefit 4-pi-related applications.

Figure 2 shows the system setup, where the light source is a 532-nm continuous-wave laser (Verdi-V5, Coherent Inc.). The laser beam is collimated and split into two beams. One is used as a seeding beam and the other is split again into reference and reading beams. To provide sufficient conjugation power, the coupling strength is increased by configuring all three beams as e-polarization in BaTiO_3_^25^. The reference and reading beams illuminate the BaTiO_3_ in counter directions. The seeding beam is focused into the sample through the P-Obj (Olympus MPLN 20x, N.A. 0.4). In the writing process, the reading beam is blocked (Fig. 2a). The backward scattering light is collected by the P-Obj and is directed to the BaTiO_3_ by the lens (L_4_). Limited by the effective area of BaTiO_3_, off-focus scattering will be blocked, so that the scattering light reaching the BaTiO_3_ mostly originates from the focal plane of the P-Obj. This structure can be considered as an effective confocal geometry. The interference fringe between the backward scattering light and the reference beam is recorded by the BaTiO_3_ and thus forms the HOPCM. In the reading process, the seeding and reference beams are blocked, and the reading beam impinges on the HOPCM to generate a conjugate wave (Fig. 2b). The conjugate wave counter-propagates along the light path of the backward scattering light. After the conjugate wave is incident on the sample and is rescattered, the scattering light behaves as if focused by an OV-Obj to inversely focus at the same focusing position of the seeding beam.

As shown in Fig. 3a, the BaTiO_3_ is at the conjugate imaging plane of the focal plane of the P-Obj. The entrance area of the BaTiO_3_ defines the field of view of the P-Obj. If the light is backward scattered from where is deviated from the focal plane of the P-Obj, most of the scattered light will be spatially filtered out by the lens L_4_ and the BaTiO_3_. To generate the inverse focus point, the backward scattering light collected by the P-Obj should come from layers deeper than the forward focus point. Therefore, we placed the lens (L_3_) in the light path of the seeding beam and shifted the focusing depth closer to the surface of the sample (Fig. 3b). The collection ratio of the backward scattering light from layers deeper than the forward focus point is thus increased. The light paths inside the media for the optical system with and without L_3_ are shown in Fig. 3c,d, respectively.

The samples used in the experiment comprised three layers, 1.2-mm agarose doped with quantum dots, scattering media, and clay. As shown in Fig. 4a, all three materials were layered in a plastic box with a fixed height. The outermost layer was the 1.2-mm agarose doped with quantum dots (Qdot 655 ITK Carboxyl Quantum Dots). The scattering property of agarose mimics the scattering behavior in bio-tissue; the fluorescence of the quantum dots can be excited by 532-nm light to reveal both the forward and inverse focusing light paths. Beneath the agarose were the scattering media. Two different scattering media, the DP9003 diffuser (Bayer Inc.) and fresh mouse ear skin, were separately used to provide the backward scattering signals. With an incident beam angled at 10° to the normal of these two scattering media, the bidirectional reflectance distribution functions (BRDFs) of the DP9003 diffuser and mouse ear skin were measured^26^, as shown in Fig. 4b,c, respectively. Haze is defined as the energy percentage of the reflected light deviating from the specular direction more than 2.5° out of the total reflected light. According to the definition, haze is 99.84% and 99.85% for DP9003 diffuser and mouse ear skin, respectively. From the intensity distribution, these two scattering media were shown to have no apparent specular reflection. This indicated that these two scattering media were highly scattering and could provide sufficient backward scattering signals required for recording. The clay at the bottom was used to adjust the thickness of the agarose.

Figure 5 illustrates the use of the OV-Obj. A camera captured the side view of the inverse focusing light path. A color filter (CF) suppressed the scattering noise of the green laser and transmitted the red fluorescent signals produced by the quantum dots (Fig. 5b,c). First, we tested the diffuser sample that used the DP9003 diffuser as the scattering medium. The attenuation coefficient of the diffuser is measured as 7.10 mm^−1^. The penetration depth with intensity dropping to 1/e is 0.14 mm. The focusing light path of the seeding beam inside the diffuser sample that was revealed by the excited fluorescent signals in the writing process is shown in Fig. 5d. The dashed lines indicate the position of the DP9003 diffuser. Figure 5e, which is a contrast enhanced image, shows the inverse focusing light path in the reading process. The focusing and inverse focusing light paths were observed to overlap precisely and have the same focal plane. Because the seeding beam was mostly backward scattered by the surface of the diffuser, no fluorescent signals were observed beneath the diffuser surface. In the reading process, the conjugate wave propagated back and was rescattered by the sample. The scattering sources deeper than the focus point emulated an OV-Obj to form the inverse focusing beam. Figure 5f,g show the focusing and inverse focusing light paths, respectively, in the skin sample that used the mouse ear skin as the scattering medium. Figure 5g is a contrast-enhanced image. The dashed lines indicate the front surface of the ear skin. The focusing and inverse focusing light paths again overlapped precisely and had the same focal plane. In contrast to the case of the diffuser sample, fluorescent signals were observed inside the ear skin during the writing process (Fig. 5f). This indicated that parts of the seeding beam penetrated to the deeper layers of the skin. Most of the backward scattering light from the deeper layers could still be collected, as long as it could propagate into the field of view of the objective. Therefore, during the reading process, the conjugate wave also penetrated to deeper layers of the skin, and the fluorescent signals were detected within the ear skin area (Fig. 5g).The detected signal actually is the conjugate wave penetrating into the skin, escaping from side face of the mouse skin and being transferred to fluorescence by the surrounding Qdots-doped agarose. Via analyzing the fluorescent intensity within the ear skin area, the penetration depth is calculated as 0.41 mm. In most types of bio-tissues, the scattering behavior of light is similar to that observed in the ear skin. Therefore, the OV-Obj in the bio-tissues is formed by a variety of scattering sources. It is known that the scattering decorrelation time for live tissues is on the level of ms^27^. Although the response time of the OV-Obj is not the topic of this study, some reports of the high-speed birefringent crystal has provided the potential solution for *in-vivo* applications^28^,^29^.

To avoid the possibility of forming a fake OV-Obj through the forward focusing of other noise in the reading process, we confirmed the existence of the conjugate wave by inserting a screen on the path of the seeding beam (Fig. 5a). The conjugate wave from the diffuser and skin sample was observed on the screen as shown in Fig. 5h,i, respectively. The yellow dashed circles indicate the original area of the seeding beam. This observation strongly proved the existence of the OV-Obj. We define the signal to background-noise ratio (SBR) on the focal plane. The SBR is defined as the mean value of the focus region divided by the mean value of the background region insides the field of view of P-Obj. From the raw data analysis, we get SBR as 2.38 and 1.90 for Fig. 5e,g, respectively. Figure 5 shows the energy of the inverse focusing is weaker than the forward focusing. In the case of 4-pi applications, amplification of optical phase-conjugator (OPC) will be useful to balance the intensity between forward focusing and inverse focusing^30^,^31^.

Fidelity is defined as the ratio of the phase-conjugate signal energy to the total energy on the observation plane. On the basis of the reciprocity theorem, the fidelity of an optical phase conjugate mirror has been clearly studied regarding whether specimens have absorption^27^,^28^. Accordingly, the fidelity of the OV-Obj can be expressed as


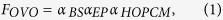


where


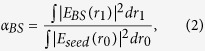



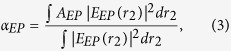


and


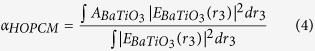


Here, α_BS_ is the fidelity related to the backward scattering ratio; and α_EP_ and α_HOPCM_ are the fidelities related to the entrance pupil of the objective and entrance area of the BaTiO_3_, respectively. In addition, E_seed_ is the electrical field at the plane immediately after the seeding beam focal plane in the sample; E_BS_ is the electrical field at the surface of the sample contributed by the backward scattering light from layers deeper than the focal plane; A_EP_ is the entrance pupil of the P-Obj; E_EP_ is the electrical field of the backward scattering light at the entrance pupil of the P-Obj; A_BaTiO3_ is the entrance area of the BaTiO_3_; and E_BaTiO3_ is the electrical field at the incident plane of the BaTiO_3_.

Equations (3) and (4) indicate that the fidelity of the OV-Obj, F_OVO_, can be further improved using an objective with a larger entrance pupil and a BaTiO_3_ with a larger entrance area. Equation (2) shows that tissues deeper than the focus point that provide strong backward scattering can benefit the fidelity of the OV-Obj.

In this research, we introduced and successfully demonstrated the concept of the OV-Obj, which provides a solution for forming an inverse focusing light path inside thick or live tissues. It can be used to increase the collection efficiency of forward-emitted signals and to fulfill the requirement for bidirectional illumination in thick or live tissues. By using the BaTiO_3_ as the HOPCM, e-polarized beams were applied to the writing and reading processes to provide sufficient coupling strength. In both the diffuser and skin samples, the inverse focusing light paths formed by the OV-Obj were clearly observed through the fluorescence of quantum dots. As expected, the light paths of the focusing and inverse focusing light overlapped precisely and had the same focal plane. A screen was set in the light path of the seeding beam to prove the existence of the conjugate beam. Because the skin sample was designed to mimic bio-tissue, the achievement of the inverse focusing in the skin sample strongly indicated the capability of this system for live applications. At the end of this paper, we indicate that the fidelity of the OV-Obj can be improved by using an objective with a large entrance pupil, a BaTiO_3_ with a large entrance area, and strong backward scattering tissues in deeper layers. We believe that an OV-Obj inside tissues enables thick-tissue and *in vivo* applications of fluorescence microscopy, second-harmonic generation microscopy, and 4-pi-related techniques.

## Methods

### Configuration of the HOPC system

**Figure 2:**
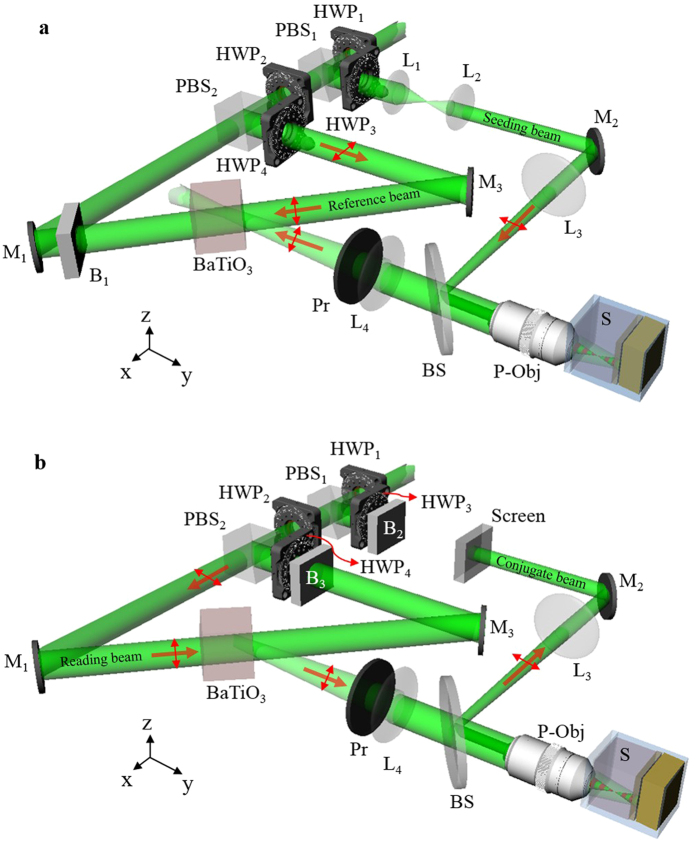
System setup based on HOPC in a BaTiO_3_. (**a**),Writing process and (**b**), reading process in the experiment. PBS, polarization beam splitter; HWP, half-wave plate; P-Obj, physical objective; BS, beam splitter; Pr, polarizer; M, mirror; B, blocker; L, lens; S, sample.

A BaTiO_3_ was used as the HOPCM. The system configuration is shown in Fig. 2. The light source was a 532-nm continuous-wave laser (Verdi-V5, Coherent Inc.). A spatial filter followed by a lens was used to collimate and expand the laser beam to 8 mm in diameter to fill the back aperture of the objective. The laser beam was split into two beams with different polarizations by a polarization beam splitter, PBS_1_. One (z-polarization) was used as the seeding beam and the other (y-polarization) was split again into reading and reference beams. Combined with a half-wave plate, HWP_1_, the relative intensity of these two beams was adjusted by rotating HWP_1_. Another half-wave plate, HWP_2_, was used to rotate the polarization of the seeding beam into x-polarization. The seeding beam was guided and reflected using a 50/50 beam splitter into an objective with a numerical aperture of 0.4, the P-Obj (Olympus MPLN 20×), and was focused into samples by the objective. The focusing depth of the seeding beam could be adjusted by inserting a lens, L_3_, before the objective. The backward scattering light was epi collected by the same objective and imaged into the BaTiO_3_ by a lens, L_4_. Before entering the BaTiO_3_, a polarizer, Pr, was used to purify the polarization of the backward scattering light to be e-polarized in the BaTiO_3_. The other beam (y-polarization), split by PBS_1_, was divided again by another polarization beam splitter, PBS_2_. One beam (z-polarization) was used as the reference beam and the other (y-polarization) was used as the reading beam. Combined with a half-wave plate, HWP_3_, the relative intensity of these two beams could be adjusted by rotating HWP_3_. The reference and the reading beams were aligned to counter-propagate to the BaTiO_3_. The reading beam with y-polarization was e-polarized in the BaTiO_3_, and the reference beam was adjusted by a half-wave plate, HWP_4_, to be e-polarized in the BaTiO_3_. During the writing process, a blocker, B, was used to block the reading beam. The interference fringes in the BaTiO_3_ were formed by the reference beam and the backward scattering light. During the reading process, the blocker was moved to block the reference beam. The conjugate wave was read out by emitting the reading beam into the BaTiO_3_.

## Additional Information

**How to cite this article**: Yu, Y.-W. *et al*. Inverse focusing inside turbid media by creating an opposite virtual objective. *Sci. Rep.* **6**, 29452; doi: 10.1038/srep29452 (2016).

## Figures and Tables

**Figure 1:**
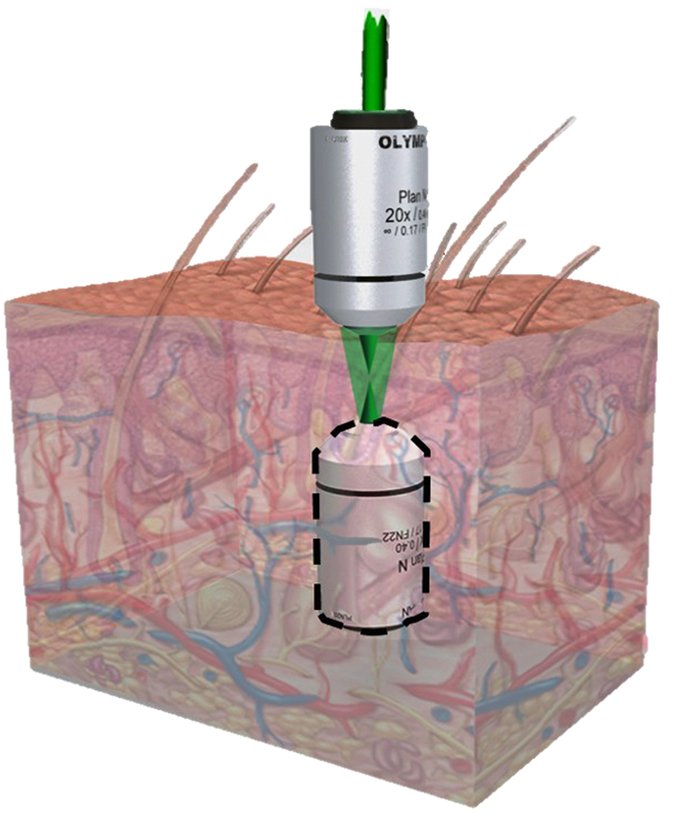
Concept of an opposite virtual objective (Dash line).

**Figure 3:**
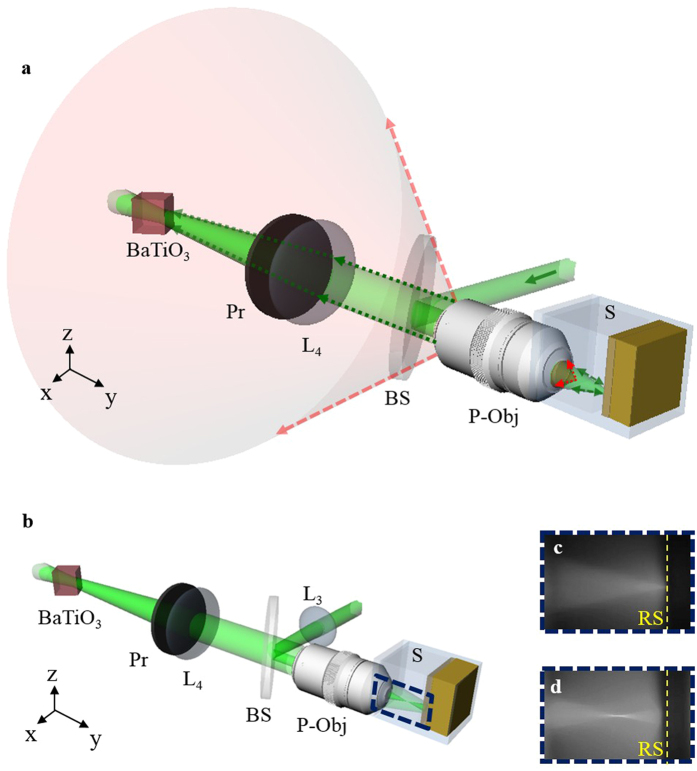
(**a**) Optical phase conjugation system. (**b**) The existence of L_3_ shifts the focus point closer to the surface of the sample. (**c**) The focusing light path without the lens (L_3_) in the region marked by the blue dashed square. (**d**) The focusing light path with the lens (L_3_) in the region marked by the blue dashed square. P-Obj, physical objective; BS, beam splitter; Pr, polarizer; L, lens; S, sample. RS, reflective sheet.

**Figure 4:**
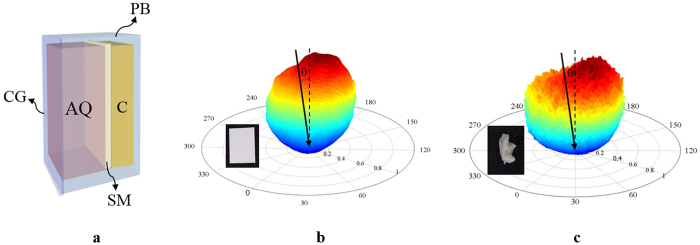
(**a**) Diagram of the sample containing three layers, agarose doped with quantum dots, scattering media, and clay. (**b**) BRDF of the DP9003 diffuser and (**c**) BRDF of the mouse ear skin with incident light angled at 10° to the normal of the sample. CG, cover glass; AQ, agarose doped with quantum dots; SM, scattering media; C, clay; PB, plastic box; θ, incident angle to the normal of the scattering media.

**Figure 5:**
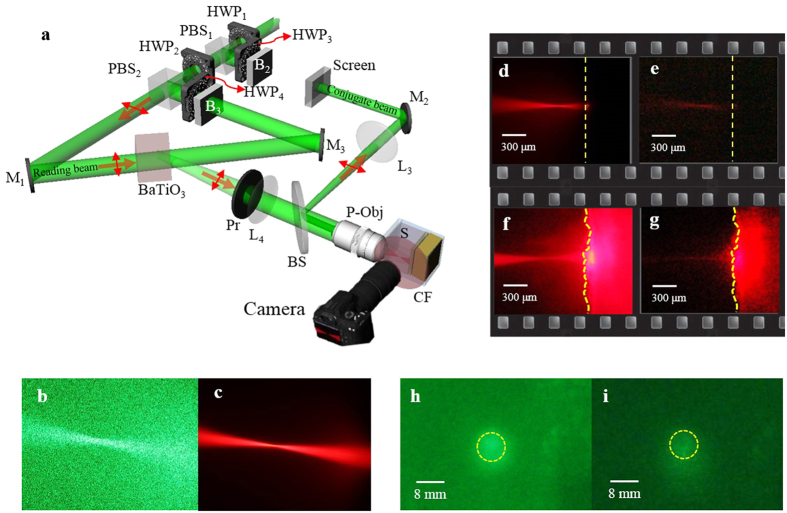
Light paths of focusing and inverse focusing in the diffuser and skin samples. (**a**) Setup; (**b**,**c**) detected images without and with the color filter; (**d**) focusing light path in the diffuser sample; (**e**) inverse focusing light path in the diffuser sample generated by the OV-Obj; (**f**) focusing light path in the skin sample; (**g**) inverse focusing light path in the skin sample generated by the OV-Obj; (**h**) conjugate wave from the diffuser sample; (**i**) conjugate wave from the skin sample. PBS, polarization beam splitter; HWP, half-wave plate; P-Obj, physical objective; BS, beam splitter; Pr, polarizer; M, mirror; B, blocker; L, lens.
